# *Nlrp2* deletion ameliorates kidney damage in a mouse model of cystinosis

**DOI:** 10.3389/fimmu.2024.1373224

**Published:** 2024-04-03

**Authors:** Marianna Nicoletta Rossi, Valentina Matteo, Francesca Diomedi-Camassei, Ester De Leo, Olivier Devuyst, Mohamed Lamkanfi, Ivan Caiello, Elena Loricchio, Francesco Bellomo, Anna Taranta, Francesco Emma, Fabrizio De Benedetti, Giusi Prencipe

**Affiliations:** ^1^ Laboratory of Immuno-Rheumatology, Bambino Gesù Children’s Hospital, Istituto di Ricovero e Cura a Carattere Scientifico (IRCCS), Roma, Italy; ^2^ Department of Science, University of Rome “Roma Tre”, Rome, Italy; ^3^ Department of Laboratories, Pathology Unit, Bambino Gesù Children’s Hospital, Istituto di Ricovero e Cura a Carattere Scientifico (IRCCS), Roma, Italy; ^4^ Division of Nephrology, Bambino Gesù Children’s Hospital, Istituto di Ricovero e Cura a Carattere Scientifico (IRCCS), Roma, Italy; ^5^ Mechanisms of Inherited Kidney Disorders Group, Institute of Physiology, University of Zurich, Zurich, Switzerland; ^6^ Laboratory of Medical Immunology, Department of Internal Medicine and Paediatrics, Ghent University, Ghent, Belgium

**Keywords:** cystinosis, inflammation, fibrosis, NLRP2, chronic kidney disease

## Abstract

Cystinosis is a rare autosomal recessive disorder caused by mutations in the *CTNS* gene that encodes cystinosin, a ubiquitous lysosomal cystine/H^+^ antiporter. The hallmark of the disease is progressive accumulation of cystine and cystine crystals in virtually all tissues. At the kidney level, human cystinosis is characterized by the development of renal Fanconi syndrome and progressive glomerular and interstitial damage leading to end-stage kidney disease in the second or third decade of life. The exact molecular mechanisms involved in the pathogenesis of renal disease in cystinosis are incompletely elucidated. We have previously shown upregulation of NLRP2 in human cystinotic proximal tubular epithelial cells and its role in promoting inflammatory and profibrotic responses. Herein, we have investigated the role of NLRP2 *in vivo* using a mouse model of cystinosis in which we have confirmed upregulation of *Nlrp2* in the renal parenchyma. Our studies show that double knock out *Ctns^-/-^ Nlrp2^-/-^* animals exhibit delayed development of Fanconi syndrome and kidney tissue damage. Specifically, we observed at 4-6 months of age that animals had less glucosuria and calciuria and markedly preserved renal tissue, as assessed by significantly lower levels of inflammatory cell infiltration, tubular atrophy, and interstitial fibrosis. Also, the mRNA expression of some inflammatory mediators (*Cxcl1* and *Saa1*) and the rate of apoptosis were significantly decreased in 4-6-month old kidneys harvested from *Ctns^-/-^ Nlrp2^-/-^* mice compared to those obtained from *Ctns^-/-^* mice. At 12-14 months of age, renal histological was markedly altered in both genetic models, although double KO animals had lower degree of polyuria and low molecular weight proteinuria and decreased mRNA expression levels of *Il6* and *Mcp1*. Altogether, these data indicate that Nlrp2 is a potential pharmacological target for delaying progression of kidney disease in cystinosis.

## Introduction

Cystinosis is a rare multisystemic autosomal recessive disorder caused by mutations in the *CTNS* gene, coding for the lysosomal cystine transporter cystinosin (1). At the kidney level, cystinosis is characterized by accumulation of intracellular cystine, formation of cystine crystals, proximal tubular dysfunction causing renal Fanconi syndrome, and progressive glomerular damage leading to kidney failure (2). Cysteamine therapy reduces lysosomal cystine accumulation and delays but does not prevent progression of kidney failure (3). To date, the exact molecular mechanisms involved in the development and progression of the disease remain incompletely understood. Beyond lysosomal cystine accumulation, other pathogenic mechanisms have been shown to play an important role in the pathogenesis of cystinosis. These include increased oxidative stress (4–6), mitochondrial dysfunction (7–9), enhanced apoptosis (10), and abnormal autophagy (5, 11, 12). Consistent with the increasing evidence showing a major role of inflammation in the development and progression of chronic kidney diseases (13, 14), several studies have shown that inflammation is also involved in the pathogenesis of kidney disease in cystinosis (15–18).

Previous results obtained by our group have pointed to a possible causative association between Nod-like receptor protein (NLRP)-mediated inflammation and progressive kidney damage in cystinosis (15). Specifically, we have shown that cystine crystals activate the inflammasome in monocytes (15); and that NLRP2 is markedly overexpressed in human cystinotic proximal tubular epithelial cells (PTEC) in a kidney biopsy obtained from a patient with cystinosis, in primary cultured PTEC and in immortalized PTEC cell lines derived from cystinotic patients (19). In PTEC, overexpression of NLRP2 upregulated proinflammatory and profibrotic as well as anti-apoptotic responses through the modulation of NF-κB activity (19). Our data were consistent with evidence demonstrating that increased renal NLRP2 expression levels are associated with acute tubular damage in human as well as in mice (20, 21).

Based on these findings, we hypothesized that upregulation of NLRP2 in cystinotic kidneys plays a role in the development of the interstitial inflammation and fibrosis, contributing to the progression to the end-stage kidney disease. In order to test our hypothesis and explore the pathogenic role of NLRP2 *in vivo*, we analyzed Nlrp2 expression in the *Ctns^-/-^* mouse model, and generated a double knock out (KO) *Ctns^-/-^ Nlrp2^-/-^* mouse model by crossing cystinotic mice with *Nlrp2^-/-^* mice.

## Materials and methods

### Generation of *Ctns* ^-/-^ *Nlrp2* ^-/-^ double-knockout mice, animal care and procedures

The C57BL/6 *Ctns* ^-/-^ mice were kindly provided by Prof. Corinne Antignac (22). C57BL/6 *Nlrp2 ^-/-^* mice have been described (23). Animal care and experimental procedures were conducted in accordance with the European 2010/63/EU directive on the protection of animals used for scientific purposes and authorized by the Italian Ministry of Health (authorization number 898/2017-PR). *Ctns* ^-/-^ *Nlrp2* ^-/-^ double-knockout mice were generated first by crossing single knockout mice to produce compound heterozygotes and then by crossing compound heterozygous mice. Genotyping of mice was carried out as previously described (23, 24). Female *Ctns* ^-/-^ and *Ctns* ^-/-^ *Nlrp2* ^-/-^ mice (n= 7 animals per group) were followed longitudinally from birth; urine samples were collected every two months, starting from the fourth month of age. Mice were then sacrificed at 6, 12 and 14 months of age. At sacrifice, kidneys were rapidly dissected and snap-frozen or processed in paraffin for further analysis.

For urine collection, mice were acclimatized in metabolic cages for 24 hours before collecting urine in the following 24 hours, in the presence of 0.1% sodium azide and 1X protease inhibitors (Thermo Scientific). Mouse weight was recorded before entering the metabolic cages. Urine analyses were performed by the veterinary laboratory Appialab Srl (Rome, Italy). Urinary glucose (mg/dL), albumin (mg/L), calcium (mg/dL), and creatinine (mg/dL) were measured with the use of HITACHI Cobas C311 (Roche Diagnostics GmbH, Mannheim, Germany). Clara cell protein 16 (CC16) urinary levels were measured using the Immunosorbent Assay kit in accordance with manufacturer’s instructions (Biomatik, Wilmington, DE).

Isolation and primary cultures of proximal tubule cells was performed as previously described (25).

### RNA isolation and quantitative real-time PCR

Total RNA was extracted from mouse kidney tissues using Trizol reagent (Ambion), and cDNA was obtained using the Superscript Vilo kit (Invitrogen). Real-time PCR assays were performed using TaqMan Universal PCR Master mix (Applied Biosystems) and the following gene expression assays: mouse *Cxcl1, Cxcl5, Il6, Ccl2, Saa1*. Gene expression data were normalized using mouse *Hprt1* (Applied Biosystems) as endogenous controls. Data are expressed as arbitrary units (AU), determined using the 2^−Δct^ method. Sybr green (Roche) was utilised to analyse *Nlrp2* expression. Primer sequences are available on request.

### Tissue histology

At sacrifice, kidneys were fixed overnight in 4% paraformaldehyde and then embedded in paraffin. Three-μm thick sections were obtained and stained with hematoxylin-eosin and Masson’s trichrome staining. Renal sections were scored blindly by Dr. Francesca Diomedi Camassei on a scale ranking 0 to 3+, to estimate the severity of tubulointerstitial changes. The following parameters were scored: immune cell infiltration, tubular atrophy, interstitial fibrosis, medullary tubular casts, cortical tubular casts, and glomerulosclerosis. A minimum of 10 fields for each kidney slide were examined. A global score was then calculated for each kidney by addition of all scores. For evaluating renal parenchyma damage, the scoring system was extrapolated from the Banff scoring system (26) and modified adding a category for very focal alterations. In our score, we considered 5 categories (instead of 4 in the Banff system): 0, no alterations (0%); 0.5, very focal alterations (1-10%); 1, mild alterations (11-25%); 2, moderate alterations (26-50%); 3, diffuse alterations (>50%).

### Evaluation of apoptosis in kidney tissue

Immunohistochemistry was performed on 2 μm thick sections obtained from formalin-fixed tissue embedded in paraffin. After dewaxing and rehydrating, heat-induced epitope retrieval was performed by boiling the slides with sodium citrate (pH 6) (Dako, Glostrup, Denmark). Endogenous peroxidase was blocked with 3% hydrogen peroxide and then with 5% bovine serum albumin (BSA). Sections were incubated overnight at 4°C with rabbit monoclonal Cleaved Caspase-3 antibody (1:300) (9661S, Cell Signaling Technology, Danvers, MA, USA). Detection of the primary antibody was performed by using the appropriate secondary biotinylated antibody (K8024) (ready to use) (Dako, Carpinteria, CA, USA) and the peroxidase DAB kit (Dako, Carpinteria, CA, USA). Counterstaining was performed with hematoxylin solution Gill2.

The entire slides were scanned and captured by NanoZoomer S60 Digital scanner, 5 random 20X fields of the subcapsular cortex for each mouse were selected using the NDP.view2 Image viewing software. The mean intensity of caspase 3 staining was measured by Image J software.

### Statistical analyses

Data are presented as mean ± SDs or as median and IQRs. Pairwise comparisons were evaluated by the Mann-Whitney *U* test. Group comparisons were performed by using 2-way Anova, followed by Tukey’s multiple comparison test.

All statistical analyses were performed using GraphPad Prism IX software. P-values lower than 0.05 were considered statistically significant.

## Results

### Cystinotic kidneys show increased inflammation markers

**Figure 1:**
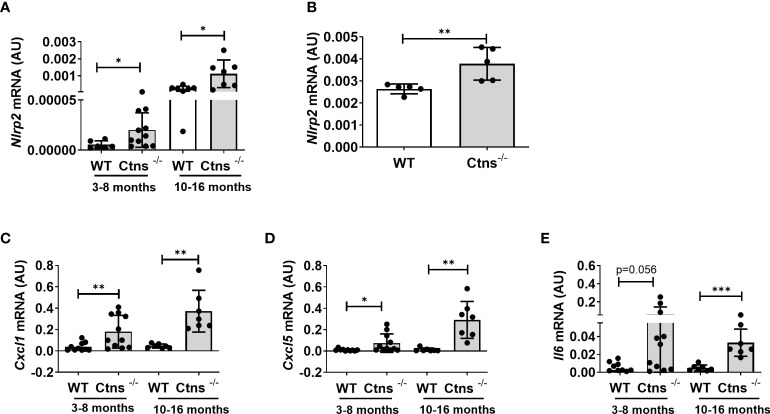
*Nlrp2* and inflammation markers are increased in cystinotic kidneys. **(A)** *Nlrp2* mRNA levels were evaluated by qPCR analysis in total kidneys from wild type (WT) and cystinotic (*Ctns^-/-^*) mice at 3-8 and 10-16 months of age and **(B)** in primary proximal tubular epithelial cells isolated from WT and *Ctns* ^-/-^ mice at 6 months of age. **(C–E)** *Cxcl1, Cxcl5 and Il6* mRNA expression levels were evaluated by qPCR analysis in total kidneys from WT and *Ctns^-/^*) mice at 3-8 and 10-16 months of age. **(A–E)** Results were obtained after normalization with the housekeeping genes *Hprt1* and are expressed as arbitrary units (AU). Differences between WT and *Ctns* ^-/-^ mice were compared using the Mann-Whitney U test. *p < 0.05; **p < 0.01; ***p < 0.001.

We previously demonstrated that the NOD like receptor protein 2 (NLRP2) is overexpressed in human cystinotic PTEC and plays a role in regulating proinflammatory and profibrotic responses. To extend our previous findings and investigate the *in vivo* role of NLRP2 in the pathogenesis of cystinosis, we first analysed the mRNA expression levels of *Nlrp2* in whole kidney lysates obtained from *Ctns* ^-/-^ and wild type (WT) mice at different ages. We found that *Nlrp2* expression levels were significantly higher in *Ctns^-/-^* kidneys compared to WT kidneys, both in the early (3-8 months of age) and in the late stage (10-16 months of age) of renal disease (**Figure 1A**). Accordingly, *Nlrp2* mRNA expression levels were significantly increased in purified primary PTECs isolated from *Ctns* ^-/-^ mice at 6 months of age, compared to PTECs from WT mice (**Figure 1B**). In addition, we analysed the expression of chemokines/cytokines that we previously demonstrated to be modulated by NLRP2 in human cells, including C-X-C motif chemokine ligand-1 (*Cxcl1*), *Cxcl5* and interleukin-6 (*Il6*). We observed significantly higher mRNA levels of *Cxcl1* and *Cxcl5* and a trend towards higher *Il6* levels (p=0.056) in *Ctns* ^-/-^ compared to WT kidneys collected at 3-8 months of age, when renal damage begins to develop in *Ctns* ^-/-^ animals (**Figure 1C**). At 10-16 months of age, when overt kidney disease is established, *Ctns* ^-/-^ kidneys showed significant upregulation of all analysed inflammatory mediators, when compared to WT kidneys (**Figures 1C–E**). Collectively, these results confirmed that *Nlrp2* expression is upregulated *in vivo* in total kidneys and in PTEC from cystinotic mice and that expression of some inflammatory chemokines/cytokines is increased in the kidney of cystinotic mice.

### Genetic deletion of *Nlrp2* partially ameliorates kidney functions in cystinotic mice

**Figure 2:**
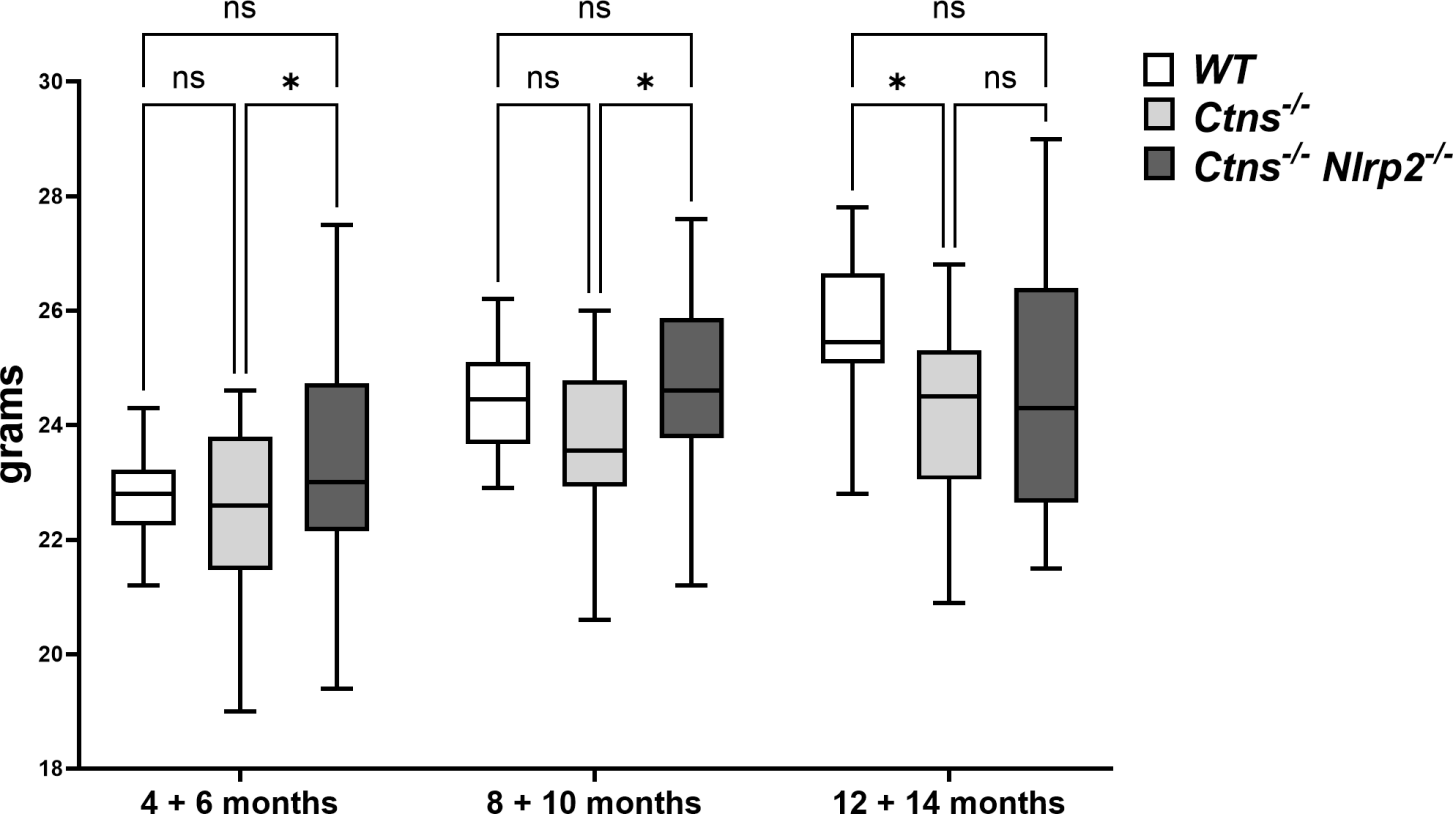
WT, *Ctns* ^-/-^ and *Ctns* ^-/-^ *Nlrp2* ^-/-^ mice body weight. WT, *Ctns^−/−^* and *Ctns* ^-/-^ *Nlrp2* ^-/-^ mice were weighed every two months from 4 to 14 months of age. Differences between groups were compared using 2-way Anova, followed by Tukey’s multiple comparison test. *p < 0.05; ns, not significant.

To investigate the *in vivo* role of *Nlrp2* in the progression of cystinotic disease, we generated a double KO mouse model by crossing C57BL/6 *Ctns* ^-/-^ mice with C57BL/6 *Nlrp2* ^-/-^ mice. Double KO animals were vital and fertile and had normal growth and lifespan. Until 10 months of age double KO mice showed a significantly greater body weight compared to *Ctns* ^-/-^ mice (**Figure 2**). Unlike *Ctns* ^-/-^ mice that, as expected (22, 27), weighted significantly less than WT animals at 12-14 months of age, *Ctns* ^-/-^ *Nlrp*2^-/-^ mice weighted similarly to WT mice at these same ages (**Figure 2**).

**Figure 3:**
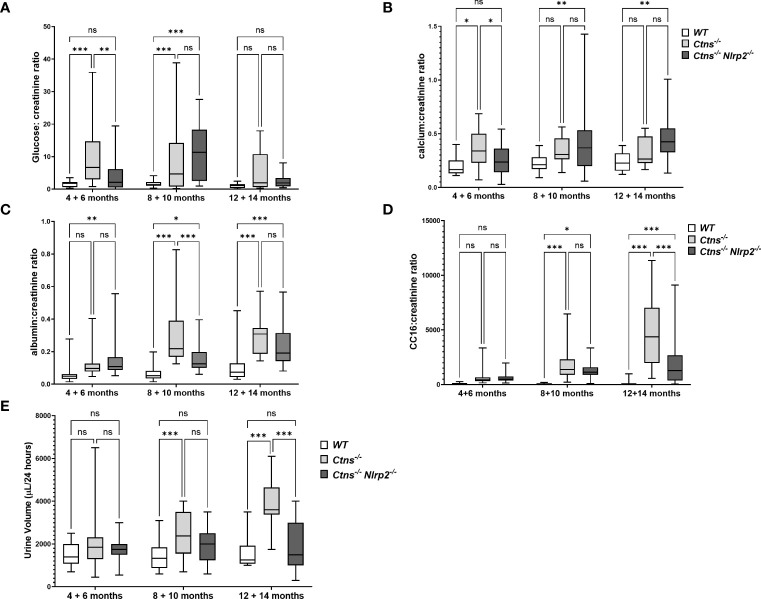
Evaluation of parameters of kidney function. **(A–D)** Glucose, calcium, albumin and Clara cell protein 16 (CC16) urinary levels were measured in WT, *Ctns^−/−^* and *Ctns* ^-/-^ *Nlrp2* ^-/-^ mice from 4 to 14 months of age and expressed as ratio against the corresponding creatinine levels. **(E)** 24h urine volume (expressed as µl/24 hours) were measured in WT, *Ctns^−/−^* and *Ctns* ^-/-^ *Nlrp2* ^-/-^ mice from 4 to 14 months of age. Differences between groups were compared using 2-way Anova, followed by Tukey’s multiple comparison test. *p < 0.05; **p < 0.01; ***p < 0.001; ns, not significant.

To investigate the development and progression of kidney disease, urine samples from *Ctns* ^-/-^ mice and *Ctns* ^-/-^ *Nlrp*2^-/-^ double KO mice were collected every two months, starting from 4 and up to 14 months of age. We evaluated urinary levels of glucose, calcium, albumin, and the low molecular weight protein Clara cell protein 16 (CC16), a marker of proximal tubular renal proximal dysfunction (28), and measured urine volumes. Results are shown in **Figure 3**. As expected, *Ctns* ^-/-^ animals developed kidney disease around 4-6 months of age. In this experimental model of cystinosis, glucosuria peaks around 10 months of age and decreases thereafter; the reason for this biphasic evolution being unclear. When comparing *Ctns* ^-/-^ to *Ctns* ^-/-^ *Nlrp*2^-/-^ mice, the onset of glucosuria and calciuria was delayed in the double KO animals (i.e. significant differences were observed at 4-6 months of age) (**Figures 3A, B**). Low molecular weight (LMW) proteinuria and polyuria were significantly lower in older animals (**Figures 3D, E**), whereas albuminuria was lower only at 8-10 months of age. Collectively, these results indicate that albeit the invalidation of the *Nlrp2* gene cannot completely rescue the Fanconi syndrome, it does result in partial improvement.

### Genetic deletion of *Nlrp2* delays the appearance of histological markers of kidney damage

Kidney samples were examined at 6, 12 and 14 months of age for the presence of renal lesions, including immune cell infiltration, tubular atrophy, interstitial fibrosis, medullary tubular dysfunction casts, cortical tubular casts and glomerulosclerosis. Our results show a significant delay in the development of kidney lesions in double KO animals with highly significant differences observed in samples obtained from animals sacrificed at 6 months, whereas no significant differences being observed at later ages.

**Figure 4:**
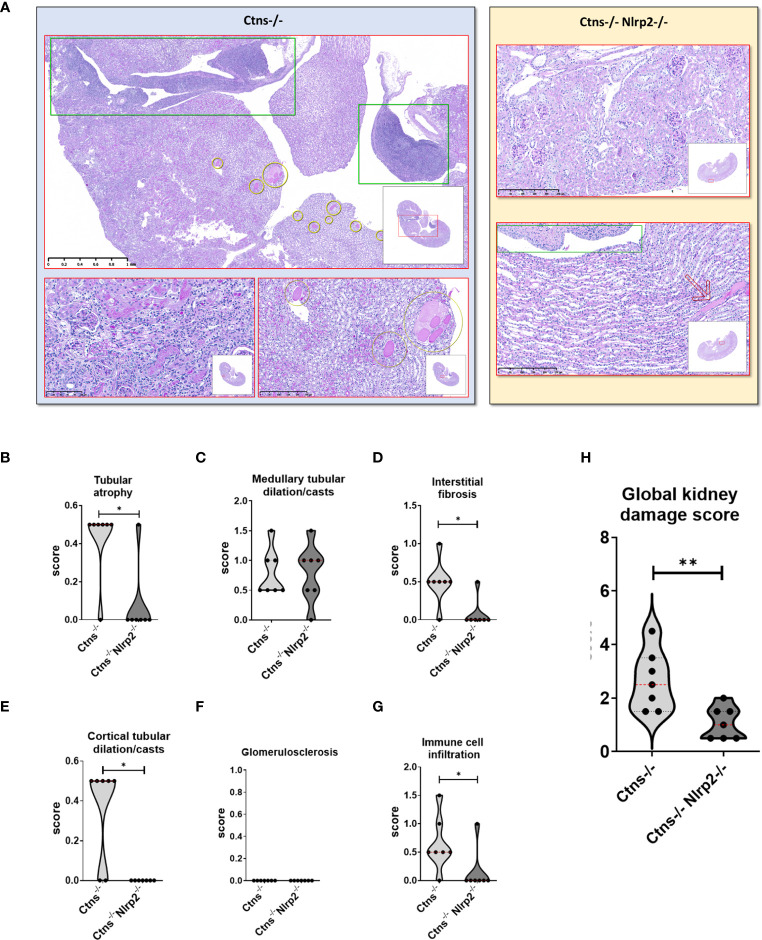
Renal histologic features of 6 months old *Ctns^-/-^* and *Ctns^-/-^ Nlrp2^-/-^* mice. **(A)** PAS stain: Renal histologic features of 6 months old *Ctns^−/−^* (left panel) and *Ctns* ^-/-^ *Nlrp2* ^-/-^ (right panel) mice. In the left panel, the *Ctns^−/−^* mice renal parenchyma: marked interstitial inflammation, especially in the medulla and renal pelvis (green squares) was observed; numerous medullary casts were present (yellow circles). At higher magnification, interstitial inflammation extended in the cortex where mild fibrosis, tubular atrophy and small hyaline casts were also evident (lower left). Medullary casts resulted unevenly distributed and showed variable size (lower right). In the right panel, *Ctns* ^-/-^ *Nlrp2* ^-/-^ mice renal parenchyma: normal glomeruli were present the cortex and no inflammation, tubular atrophy or fibrosis were observed (up). Medulla showed preserved tubular structure and distribution. Renal pelvis was not inflamed (green square). Very occasional medullary casts were present (red arrow). Renal sections were scored using a scale ranging from 0 to 3 (0= absent damage - 3= very marked damage); tubular atrophy **(B)**, medullary tubular dysfunction casts **(C)**, interstitial fibrosis **(D)**, cortical tubular casts **(E)**, glomerulosclerosis **(F)** and immune cell infiltration **(G)** were evaluated. **(H)** The sum of all scores were used to calculate the global kidney damage score. Differences between *Ctns^-/-^* and *Ctns^-/-^ Nlrp2^-/-^* mice were compared using the Mann-Whitney U test *p < 0.05; **p < 0.01.

**Figure 5:**
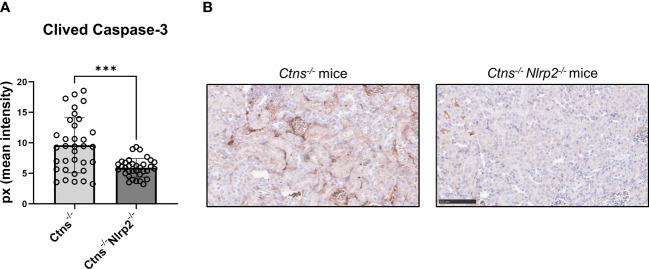
Apoptosis rate in kidney of *Ctns^-/-^* and *Ctns^-/-^ Nlrp2^-/-^* mice. **(A)** Immunohistochemistry of cleaved Caspase-3 was performed in kidney sections from *Ctns^-/-^* (n= 7) and *Ctns* ^-/-^ *Nlrp2* ^-/-^ (n= 7) mice at 6 months of age. For each section, 5 fields were acquired. Total staining intensity were analysed with imageJ Software and reported as pixel mean intensity. **(B)** Representative image of kidney sections from *Ctns^-/-^* (left) and *Ctns* ^-/-^ *Nlrp2* ^-/-^ (right). Scale bar=100μm. Differences between WT and *Ctns* ^-/-^ mice were compared using the Mann-Whitney U test. ***p < 0.001.

**Figure 6:**
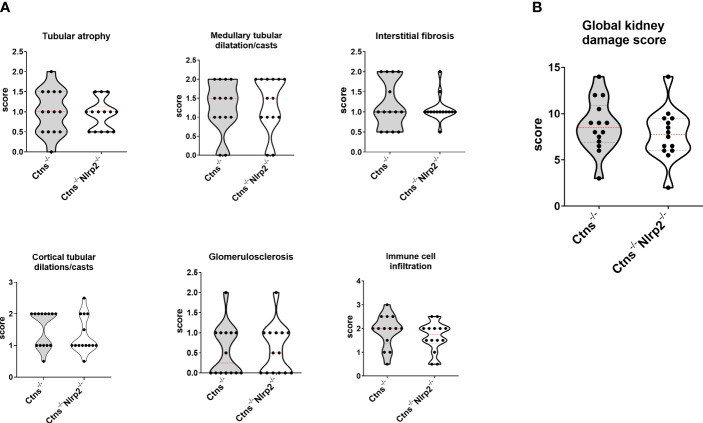
Renal histologic features of 12/14 old months *Ctns^-/-^* and *Ctns^-/-^ Nlrp2^-/-^* mice. **(A)** Kidney sections from *Ctns^-/-^* and *Ctns* ^-/-^ *Nlrp2* ^-/-^ mice at 12/14 months of age were stained with haematoxylin-eosin and Masson’s trichrome staining. Renal sections were scored using a scale ranging from 0 to 3, (0= absent damage - 3= very marked damage), tubular atrophy, medullary tubular dysfunction casts, interstitial fibrosis, cortical tubular casts, glomerulosclerosis and immune cell infiltration were evaluated. **(B)** The sum of all scores were used to calculate the global kidney damage score. Differences between *Ctns^-/-^* and *Ctns^-/-^ Nlrp2^-/-^* mice were compared using the Mann-Whitney U test.

As expected, *Ctns* ^-/-^ mice showed evidence of kidney tissue damage at 6 months of age (**Figures 4A–G**). By contrast, double KO mice showed a marked attenuation of tubular atrophy (p=0.029) and interstitial fibrosis (p= 0.025), and significantly less cortical tubular casts (p=0.021), (**Figures 4A–G**). Inflammatory infiltrates were almost absent in the double KO animals (1/7 vs. 6/7 - p=0.029, Fisher’s exact test) (**Figure 4G**). The global kidney damage score confirms that genetic invalidation of *Nlrp2* protected against the early development of kidney parenchymal lesions (p=0.009), (**Figure 4H**). The apoptosis rate was assessed by immunohistochemical staining for cleaved Caspase-3 and was also significantly decreased in double KO mice (p<0.001), (**Figures 5A, B**). By contrast, analyses of *Ctns* ^-/-^ and *Ctns* ^-/-^ *Nlrp*2^-/-^ kidneys collected at 12 and 14 months of age showed severe renal lesions in all samples, without significant differences between the two genetic models (**Figure 6A**). The global kidney damage score was significantly increased compared to 6-month old animals and was not attenuated by *Nlrp2* invalidation (**Figure 6B**).

**Figure 7:**
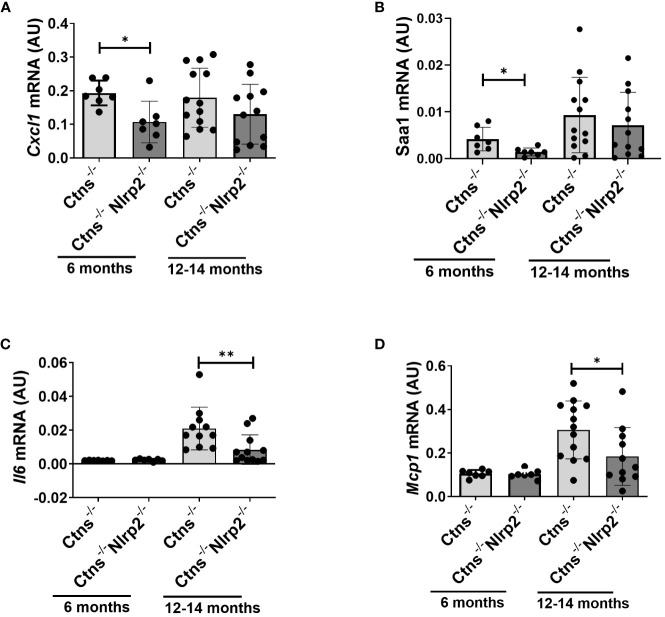
Inflammatory markers are decreased in kidney of *Ctns^-/-^ Nlrp2^-/-^* mice. **(A–D)** *Cxcl1, Saa1 Il6, and Mcp1*, mRNA expression levels were evaluated by qPCR analysis in total kidneys from *Ctns^-/-^* and *Ctns* ^-/-^ *Nlrp2* ^-/-^ mice at 6 and 12-14 months of age. Results are obtained after normalization with the housekeeping gene *Hprt1* and are expressed as arbitrary units (AU). Differences between *Ctns* ^-/-^ and *Ctns* ^-/-^ *Nlrp2* ^-/-^ mice were compared using the Mann-Whitney U test. *p < 0.05; **p < 0.01.

Lastly, we evaluated in whole kidney lysates the mRNA expression levels of proinflammatory mediators, including *Cxcl1*, *Il6*, monocyte chemoattractant protein-1 (*Mcp1*), and serum amyloid A (*Saa1*) in 6- and 12/14-months old samples. *Cxcl1* and *Saa1* mRNA levels were significantly lower in *Ctns* ^-/-^ *Nlrp2* ^-/-^ mice at 6 months of age compared to *Ctns^-/-^* mice (**Figures 7A, B**), while comparable levels of *Il6* and *Mcp1* were observed (**Figures 7C, D**). At 12-14 months, the mRNA expression of *Il6* and *Mcp1* was attenuated in the double KO animals (**Figures 7A, B**), whereas expression of *Cxcl1* and *Saa1* increased to similar levels (**Figures 7C, D**).

Taken together, results of this study indicate that the genetic ablation of the *Nlrp2* gene delays the onset of kidney disease in *Ctns* ^-/-^ mice by reducing inflammatory infiltration, interstitial fibrosis, tubular lesions and apoptosis.

## Discussion

We have previously reported that the NOD-like receptor protein NLRP2 is upregulated in a human cystinotic kidney and in PTECs, and that stable exogenous upregulation of NLRP2 in control PTEC induces proinflammatory and profibrotic responses (19). In the present study, we showed that *Nlrp2* is upregulated in kidney samples and PTECs obtained from the C57BL/6 *Ctns* ^-/-^ mouse model. Consistent with previous findings (15, 17, 22), we have confirmed upregulation of several inflammatory mediators, including those modulated by NLRP2, namely *Il6*, *Cxcl1* and *Cxcl5* at early and late stages of the kidney disease.

Building on these findings, we next generated *Ctns* ^-/-^ *Nlrp2* ^-/-^ double KO mice to dissect the contribution of *Nlrp2* in the development and progression of kidney disease in cystinosis. We observed that although genetic ablation of *Nlrp2* did not abolish the development of Fanconi syndrome or renal parenchymal lesions, it did delay it. When comparing animals analysed at 4-6 months, double KO animals showed less glucosuria and calciuria, and later in life less polyuria and LMW proteinuria. The urinary biochemical analyses were supported by the histological data showing markedly preserved renal parenchymal tissue at 6 months of age, including a lower degree of inflammatory cell infiltration, tubular atrophy, cortical and tubular dilatations/casts and interstitial fibrosis. These findings were further supported by decreased expression of some inflammatory mediators, such as *Cxcl1* and *Saa1*, and a lower apoptosis rate in kidney samples of *Ctns* ^-/-^ *Nlrp2* ^-/-^ double KO mice obtained at 6 months of age. However, when we analysed animals at 12/14 months of age, the severity of histological lesions increased to the same extent in all animals, despite double KO animals exhibiting less polyuria and LMW proteinuria and displaying lower mRNA expression of *Il6* and *Mcp1*. Altogether, these results demonstrate a role for *Nlrp2* in the progression of kidney disease in the *Ctns* ^-/-^ mouse model, and show that inhibition of this NOD-like receptor family member can partially delay disease onset.

The mode of action through which *Nlrp2* deficiency induces these effects has not been investigated at the molecular level. However, the absence of significant inflammatory infiltrates and interstitial fibrosis in most 6-months old double KO animals strongly suggests that *Nlrp2* is involved in modulating inflammatory and profibrotic responses in the kidneys of *Ctns* ^-/-^ mice at the early stages of the disease. These *in vivo* observations are in full agreement with our previous *in vitro* data, demonstrating in PTEC that *NLRP2* overexpression upregulates the expression and production of many proinflammatory and profibrotic genes by modulating the activity of the transcription factor NF-κB (19). Indeed, it has been widely demonstrated that the local production of inflammatory mediators by hematopoietic and non-hematopoietic cells, including *Cxcl1*, *Saa1*, *Il6* and *Mcp1*, which we found modulated in cystinotic kidney by *Nlrp2* deletion, plays an important role in promoting immune and inflammatory responses which, in turn, may contribute to the exacerbation of kidney damage (29, 30). Contrary to results obtained *in vitro* showing a role for NLRP2 in promoting anti-apoptotic responses in PTECs, *Nlrp2* deficiency in cystinotic mice induced a significant decrease in renal apoptotic rate, as assessed by cleaved Caspase-3 staining. In the literature, NLRP2 has been shown to either induce or decrease apoptosis, depending on cell or tissue type and on the model source (human or mouse) (21, 31–33). Therefore, the discrepancy that we observed could be related to a different behaviour of NLRP2 in mice compared to humans or *in vitro* compared to *in vivo*. However, recently Nlrp2 has been shown to have other functions, in addition to regulating NF-κB and apoptotic processes, including initiating inflammasome activation (34), modulating anti-viral responses by binding TBK-1 (35), and modulating DNA methylation at imprinted loci (36, 37). We cannot therefore exclude that Nlrp2 may regulate additional pathways involved in the progression of cystinosis; further studies are needed to better understand the precise role of Nlrp2 in renal pathophysiology and specifically in cystinosis.

In conclusion, our data add to the growing body of evidence demonstrating a role for inflammation in the development and progression of kidney disease in cystinosis (38). The activation of multiple inflammatory pathways and their interplay with other mechanisms involved in the pathogenesis of cystinosis, including increased oxidative stress, autophagy, mechanisms of cell death, and tissue fibrosis, have been reported (15–17, 19). Accordingly, treatments of cystinotic mice with drugs that have a primary or secondary anti-inflammatory effect, such as genistein, the IL-1 blocking agent anakinra or rapamycin, have been demonstrated to improve the cystinotic phenotype in experimental models (12, 18, 39, 40). Cysteamine *per se*, in addition to promoting cystine efflux from lysosomes, has been shown to exert anti-oxidative and anti-inflammatory effects (41–44). However, these therapeutic interventions cannot completely rescue the renal phenotype, but only ameliorate kidney disease, similarly to the genetic invalidation of *Nlrp2*. These results highlight the complexity of the physiopathology of cell dysfunction in cystinosis and the multiplicity of activities that the cystinosin protein plays in cells, beyond its function as a cystine/H^+^ symporter.

A limitation of our study was the use of a whole-body double KO model for *Ctns* and *Nlrp2*. Therefore, we cannot exclude that amelioration/delay in kidney damage we documented in *Ctns^-/-^ Nlrp2^-/-^* mice are indirect as changes in other tissues, or cells other than PTEC, may have an effect on kidney disease progression. Generation of a PTEC-specific *Nlrp2* knockout mouse would have allowed us to understand the exact role of Nlrp2 in PTEC and in the pathogenesis and progression of kidney disease in cystinosis.

In conclusion, our data show a partially protective effect of *Nlrp2* inhibition in the cystinotic mouse model and provide additional rationale for testing anti-inflammatory strategies in addition to cysteamine to delay progression of kidney disease in patients with cystinosis.

## Data availability statement

The raw data supporting the conclusions of this article will be made available by the authors, without undue reservation.

## Ethics statement

The animal studies were approved by Animal care and experimental procedures were conducted in accordance with the European 2010/63/EU directive on the protection of animals used for scientific purposes and authorized by the Italian Ministry of Health (authorization number 898/2017-PR). The studies were conducted in accordance with the local legislation and institutional requirements. Written informed consent was obtained from the owners for the participation of their animals in this study.

## Author contributions

MNR: Conceptualization, Data curation, Formal analysis, Investigation, Methodology, Writing – original draft, Writing – review & editing. VM: Conceptualization, Data curation, Formal analysis, Investigation, Methodology, Writing – original draft, Writing – review & editing. FD-C: Data curation, Investigation, Methodology, Writing – original draft, Writing – review & editing. EDL: Data curation, Investigation, Software, Writing – original draft, Writing – review & editing. OD: Data curation, Investigation, Writing – original draft, Writing – review & editing. ML: Investigation, Methodology, Writing – original draft, Writing – review & editing. IC: Data curation, Methodology, Writing – original draft, Writing – review & editing. EL: Investigation, Methodology, Writing – original draft, Writing – review & editing. FB: Data curation, Methodology, Writing – original draft, Writing – review & editing. AT: Data curation, Methodology, Writing – original draft, Writing – review & editing. FE: Investigation, Writing – original draft, Writing – review & editing, Data curation. FDB: Investigation, Methodology, Writing – original draft, Writing – review & editing. GP: Conceptualization, Data curation, Formal analysis, Funding acquisition, Investigation, Methodology, Project administration, Resources, Supervision, Validation, Visualization, Writing – original draft, Writing – review & editing.
